# People and Pets: Good for Each Other?

**DOI:** 10.3390/ani15142007

**Published:** 2025-07-08

**Authors:** Lindsay E. Murray

**Affiliations:** Division of Psychology, University of Chester, Chester CH1 4BJ, UK; l.murray@chester.ac.uk

## 1. Why Is This Subject Important?

Why is it timely to gather a collection of papers on the relationships between people and their companion animals? Recently, the human population has undergone an existential crisis in the wake of the COVID-19 pandemic. Simultaneously, due to climate change, the planet we live on is experiencing a crisis of extinction and a loss of biodiversity. Anthropogenic impacts play a significant role in all of these phenomena. As humans, we need to learn how to live alongside wildlife and share space in a knowledgeable and compassionate way that works to conserve what we still have and bring back what we have lost. While radical changes are needed to create a new and improved Anthropocene [1], the companion animals that we choose to live with are a good place to start when seeking to better understand the intricacies and complexities of human–animal interactions and relationships. Recent estimates put the world population of pets at one billion [2]. It is harder to obtain the specific number of pets that have been abandoned or surrendered to shelters, but one estimate puts this number at 6.5 million pets per year [3], and PETA estimates that there is a staggering population of 70 million stray dogs and cats without a home in the US alone [4]. This collection brings together papers that explore some of the factors that affect people’s decisions to form, maintain and sometimes relinquish relationships with companion animals.

## 2. Connectedness to Nature

One positive aspect that emerged from the pandemic was an increased opportunity for people to connect with nature [5]. People led restricted lives, and their daily walk was a chance to get outside and appreciate nature through an altered lens. There were many documented examples of animals being observed where they had not been noticed before, which may have been because the animals felt more confident in emerging due to more quiet surroundings, and/or because they had always been there, but people had not noticed them. People’s attitudes seemed to shift. The changing nature of our relationships with animals can be seen, for example, in the reduction in hunting and the use of animals in the US, alongside an increase in the enjoyment of wildlife-watching [6]. However, a disconnection with nature still exists [7], and people often feel less positive about encountering wildlife in their own home territories [8]. The tension between connection and disconnection can be challenging, and often reflects institutional pressures and regulations stifling professional and individual creativity and passions; for example, teachers can design a curriculum to inspire engagement with nature, yet the schools they work in may prioritize desk-based traditional learning to satisfy standard achievement metrics.

## 3. Media Influence

In an age of increasing visual stimulation and communication through the media, and especially social media, ‘videophilia’ [9] has arguably begun to replace ‘biophilia’ [10], that is, humans’ basic affinity for nature. To fix the world we live in, we need to nurture and strengthen the reconnection to nature that was facilitated by the pandemic. Science and the media can influence public perceptions; for example, the documentary My Octopus Teacher [11] did this by highlighting the sophisticated cognition and sensitivity of one particular octopus, resulting in the subtle development of informed sympathy for this species. There are already many ‘popular’ books for sale that touch on the subject of homeless dogs and cats, and at least one, A Streetcat Named Bob [12], has also been made into a film that has touched a wide audience. Rescue organizations like the RSPCA and Shelter Animals Count run campaigns related to animal homelessness and responsible pet ownership, but we need more ammunition to really change things for the better.

## 4. Effects of the Pandemic

When the pandemic started, there was media portrayal of an increase in the number of people adopting pets, especially dogs and cats, but the actual data do not support this rosy picture (Murray et al., this collection). The media liked the narrative of people with more time on their hands deciding to bring an animal companion into their homes, and, indeed, many people did buy puppies, often at grossly inflated prices [13,14]; however, most did not adopt shelter animals. A positive image is frequently portrayed of how human–pet relationships can provide humans with major benefits in terms of their physical and mental health [15]; however, pets also come with expenses, which may be prohibitive for some in the current cost of living crisis, and the animals have their own carbon footprints too [16]. Many pet-related behaviours did change during the pandemic: people searched the internet more for content involving charismatic and cute animals, including searching for particular potential pets [17], but this was not necessarily followed through with adoption. The influence of watching online videos on real pet ownership is the focus of one of the papers in this collection (Zhang et al., this collection).

## 5. A Pet Crisis

Perhaps misguided by media portrayals and mediated by a lack of research into what pet ownership actually involves (Mead et al., this collection), far too many people make the decision to abandon their companion animals or return them to shelters. The particular background of an individual animal may not be taken into account, and this can drastically affect its behaviour. Unless new owners are willing and able to take action to properly support these animals in their transition to a new home, undesirable behaviours can persist, and this proves to be one of the main reasons for the relinquishment of animals (Murray et al., Wilson et al., this collection). Currently, the media are reporting a ‘pet crisis’ as shelters struggle with a huge influx of unwanted pets, possibly resulting from the pandemic and the cost of living crisis; however, once again, it is not yet clear whether these triggers and outcomes are linked in quite the same way as is portrayed in the media (Murray et al., this collection).

## 6. Anthropocentrism

Most of the problems that occur in human–animal relationships result from humans themselves. Our domination of the planet and our anthropocentrism has caused problems for companion animals, including a lack of knowledge about different species and their needs, and a misguided belief that all animals of a given species or breed are largely the same when, in fact, individual differences are extensive and highlight the need for clear expectations around care and compatibility. The papers in this collection span countries including the UK, Austria, Germany and China, all presenting a clear picture of just some of the problems that need addressing.

## 7. Knowledge and Compassion

Science is full of biases, and this area is no exception. When we think of pets, we may primarily consider dogs (the focus of many of the papers in this collection) and cats, and then smaller mammals, and perhaps some birds and fish. Reptiles are certainly relatively overlooked, and invertebrates even more so, yet the welfare of all of these taxa is important (and the latter account for most of the world’s species). As Dame Jane Goodall famously said, only if we understand, will we care; only if we care, will we help; and only if we help, will animals be saved. However, information is lacking for many species, and humans tend to show a preference for popular and attractive animals, certainly when considering animals for companion pets. Animals that are far less familiar to us are likely to remain so, and their welfare is thus more likely to be neglected. As new research raises awareness of more widespread sentience, sensitivities and cognisance in species such as bumblebees [18] and fish [19], we are developing a deeper understanding of the importance of our relationships with animals. Surely, hand in hand with this, comes an increased responsibility to promote optimal welfare for all species? We consider our pets to be members of our family, so let us ensure that they are treated as such.
